# Reduced Floxuridine Dose Limits Hepatobiliary Toxicity Without Negatively Impacting Survival After Resection of Colorectal Cancer Liver Metastases

**DOI:** 10.1245/s10434-025-17783-y

**Published:** 2025-07-22

**Authors:** Kevin P. Labadie, Darrell Fan, Dana A. Dominguez, Paul Wong, Elizabeth Meshkin, Peter Vien, S Cecilia Lau, Jonathan Kessler, Lawrence D. Wagman, Yuman Fong, Marwan G. Fakih, Laleh G. Melstrom

**Affiliations:** 1 Division of Surgical Oncology, Department of Surgery, City of Hope National Medical Center, Duarte, CA USA; 2 Department of Pharmacy Services, City of Hope National Medical Center, Duarte, CA USA; 3 Division of Interventional Radiology, Department of Diagnostic Radiology, City of Hope National Medical Center, Duarte, CA USA; 4 Department of Medical Oncology & Therapeutics Research, City of Hope National Medical Center, Duarte, CA USA

## Abstract

**Background:**

Hepatic arterial infusion (HAI) of floxuridine is an effective adjuvant therapy for colorectal liver metastases (CRLM) following complete resection, but its use is limited by hepatobiliary toxicity. Dosing characteristics, factors associated with dose reductions, and the impact of starting dose on hepatobiliary toxicity and survival in the adjuvant setting are poorly described.

**Methods:**

From 2015 to 2024, patients who received adjuvant HAI floxuridine after complete CRLM resection at City of Hope were included. Hepatobiliary toxicity and oncologic outcomes of standard (0.12 mg/kg/day) versus reduced (0.08 mg/kg/day) dosing were compared. Extent of hepatobiliary toxicity, factors associated with dose reductions, hepatic recurrence-free survival (H-RFS), and overall survival (OS) were examined.

**Results:**

Seventy-one patients were included (median age 52). Dosing information was available for 69 patients with 40 (58%) receiving the standard dose and 29 (42%) receiving the reduced dose. Patients receiving the standard dose had significantly higher rates of hepatobiliary toxicity, including increased transaminases, alkaline phosphatase, and bilirubin levels. Biliary sclerosis requiring endoscopic intervention occurred only in the standard-dose group (*n* = 4, 10%). Dose reductions were more frequent in the standard-dose group (82% vs. 53%, *p* = 0.04). Despite lower cumulative floxuridine exposure, the reduced dose did not compromise oncologic outcomes.

**Conclusions:**

A reduced starting floxuridine dose (0.08 mg/kg/day) is associated with lower hepatobiliary toxicity without compromising survival after complete resection of CRLM. These findings support adopting the reduced dose as a new standard in the adjuvant treatment of resected CRLM.

**Supplementary Information:**

The online version contains supplementary material available at 10.1245/s10434-025-17783-y.

A many as 154,000 patients will be diagnosed with colorectal cancer in the United States in 2025.^1^ The liver remains the most common site of metastases, with approximately 50% of patients developing liver metastases during their lifetime.^2–4^ For patients with resectable colorectal liver metastases (CRLM), multimodal therapy, including surgical resection, is the only curative option and can achieve long-term disease-free survival in well selected patients.^5^ However, recurrence in the remnant liver is common and significantly limits long-term survival.^4,6^ Therefore, mitigating recurrence is important with effective adjuvant therapies.

Adjuvant hepatic arterial infusion (HAI) therapy reduces recurrence and prolongs survival in select patients after complete resection of CRLM.^7–10^ Floxuridine is the preferred HAI agent owing to its high first-pass hepatic metabolism, enabling concentrations 400 times higher than systemic administration with minimal systemic adverse effects.^11,12^ However, floxuridine is associated with significant dose-limiting hepatobiliary toxicity, including chemical hepatitis and biliary sclerosis.^13,14^ Familiarity with dosing, the co-administration of corticosteroids and omission of concurrent antivascular endothelial growth factor receptor therapy (i.e., bevacizumab) have mitigated toxicities and contributed to a recent increase in the utilization of HAI in the treatment of CRLM.^15,16^ However, the current standard floxuridine dose of 0.12 mg/kg/day remains hepatotoxic for most patients and has led to modified dosing regimens in the treatment of unresectable CRLM.^17^ To our knowledge, a reduced dosing strategy has not been evaluated in the adjuvant setting.

The purpose of our study is to examine the hepatobiliary toxicity and oncological outcomes associated with standard (0.12 mg/kg/day) and reduced (0.08 mg/kg/day) dosing of HAI floxuridine in the adjuvant treatment of CRLM.

## Methods

### Patients and Study Definitions

We conducted a retrospective cohort study of all patients who received adjuvant HAI floxuridine therapy following complete hepatic resection for CRLM between January 2015 and May 2024 at City of Hope National Medical Center. Exclusion criteria included patients with unresectable CRLM, a symptomatic primary tumor (e.g., obstruction, perforation, hemorrhage), staged hepatectomy, or postoperative residual hepatic disease. The data collected included patient demographics, operative details, tumor pathology, and molecular profiles. This study was reviewed and approved by the City of Hope National Medical Center Investigational Review Board (IRB#20298) and was conducted in accordance with the Declaration of Helsinki.

Perioperative computed tomography and magnetic resonance imaging were utilized to identify and measure hepatic metastases. Primary tumor staging was based on guidelines from the Eighth Edition of the American Joint Committee on Cancer Staging Manual ^18^. A Tumor Burden Score and Clinical Risk Score was calculated for all patients as previously described.^19,20^ Hepatic resections were defined by the Brisbane classification, with major resection consisting of hepatectomy involving 3 or more contiguous liver segments.^21^ Extrahepatic disease was defined as the presence of metastatic sites outside the liver. Postoperative complications were graded according to the Clavien-Dindo classification of surgical complications, and readmission rates were monitored for up to 30 days following discharge after surgery.^22^ Hepatic recurrence-free survival was defined as the time from surgery to the presence of radiographic recurrence of disease in the liver. Overall survival was defined as the time from surgery to the date of death from any cause.

### Hepatic Arterial Infusion Floxuridine Dosing

Based on institutional protocols, patients received HAI floxuridine concurrently with heparin, dexamethasone, and normal saline, up to a total infusion volume of 20 mL for Medtronic system or 30 mL for the Codman/Intera system ideally within 6 weeks of surgery. Additionally, systemic chemotherapy without bevacizumab was co-administered biweekly, most commonly dose-reduced 5-FU, leucovorin, and irinotecan (FOLFIRI). Patients who had HAI pump placement before 2018 received the Codman 3000 HAI pump (Johnson & Johnson, NJ). After 2018, patients received either the Medtronic Synchromed II (Medtronic, MN) or Intera 3000 pump (Intera Oncology, Boston, MA).

Hepatic arterial infusion of floxuridine was administered monthly, with each cycle infused over 14 days. The starting dose of floxuridine was selected at the discretion of the medical oncologist and was either 0.06, 0.08, 0.1, or 0.12 mg/kg/day. The standard starting dose was initially 0.12 or 0.1 mg/kg/day based on previously published data.^23^ Owing to the observed increase in hepatobiliary toxicity of 0.12 mg/kg/day, 0.08 mg/kg/day was preferentially selected over time and eventually became the new standard starting dose that was uniformly delivered. In patients with poor liver function at the time of treatment initiation, 0.06 mg/kg/day was sparingly used. For comparative analysis in this study, the standard dose was defined as 0.12 or 0.1 mg/kg/day while the reduced dose was defined as 0.08 or 0.06 mg/kg/day.

The intended adjuvant treatment intensity was 4 cycles of the starting floxuridine dose. The total cumulative floxuridine dose was defined as the total amount of floxuridine delivered during the 4 cycles of therapy. The cumulative dose reduction was defined as the difference between the intended and the actual dose of floxuridine over 4 cycles. The dose intensity represents the dose of drug (mg/kg) per week of therapy. The relative dose intensity (RDI) is represented as the fraction of the intended dose received (RDI = 100 × [floxuridine dose received (mg/kg/week) ÷ intended floxuridine dose (mg/kg/week)].

Floxuridine dose reductions were conducted at the discretion of medical oncologist. Dose reductions were conducted based on interpretation of laboratory values for alkaline phosphatase, alanine aminotransferase (ALT), aspartate transaminase (AST), and bilirubin following protocols that have been described elsewhere in addition to patient reported symptoms, such as abdominal pain.^24^ A dose reduction was defined as a decrease in the floxuridine dose at any time during therapy for any cause. Multiple dose reductions in a single patient were considered as a single dose reduction. Once a dose was reduced, it was not increased subsequently during treatment and no doses were escalated at any point during treatment. Serial hepatic enzymes were examined, and the highest value identified during active treatment was included in the analysis. Biliary sclerosis was defined as obstructive hyperbilirubinemia with concomitant biliary stricture requiring endoscopic stent placement that was not due to malignancy or other identifiable cause.

### Statistical Analyses

All categorical variables were expressed as percentages, while continuous variables were presented as medians with either the 25th and 75th quartiles or range, as specified. Comparisons of categorical groups were performed by using Fisher’s exact test, whereas comparison of continuous variables were conducted using the unpaired Student’s *t*-test for data with a Gaussian distribution and the Mann-Whitney test for data with a non-Gaussian distribution. Multivariate analyses were performed on STATA software and remaining statistical analyses were performed using Graphpad Prism version 10.0.0 (GraphPad Software, San Diego, CA). Kaplan-Meier method and log-rank tests were used to compare overall survival and hepatic recurrence-free survival between groups.

## Results

Seventy-one patients received adjuvant HAI floxuridine after complete resection of CRLM (Table 1). The primary colorectal tumors were predominantly left-sided (88%, n = 63) and KRAS wild-type (61%, n = 44). Patients had extensive liver metastases with elevated Clinical Risk and Tumor Burden score (Table 1; Supplemental Fig. 1). Seventy patients (99%) received neoadjuvant chemotherapy for a median of 8 cycles (interquartile range 6–11) prior to hepatectomy. Most patients underwent partial hepatectomy with concurrent hepatic ablation (70%, n = 50) with complete treatment of their CRLM at the time of pump placement. Adjuvant floxuridine was initiated at a median of 4.4 weeks postoperatively (range 2–12 weeks).

**Table 1:** Baseline patient and disease characteristics

Characteristic	All patients N = 71	Standard dose n = 40	Reduced dose n = 29	*p*
Age (median, range)	52 (24–76)	49 (34–76)	53.5 (38–72)	0.03
Female sex (n, %)	31 (44%)	18 (46%)	12 (40%)	0.63
Comorbidities (n, %)	55 (77%)	29 (74%)	24 (80%)	0.77
BMI (median, IQR)	25 (23–31)	25 (23–31)	25 (22–31)	0.64
*Location of primary tumor*				
Right colon	8 (11%)	4 (10%)	4 (13%)	0.72
Left colon	63 (88%)	35 (90%)	26 (87%)	0.72
CEA at dx (ng/mL, median, IQR)	54 (8–99)	21 (8–72)	29 (4–88)	0.89
Synchronous presentation (n, %)	61 (86%)	33 (83%)	26 (90%)	>0.99
pT3/4 primary tumor (n, %)	53 (75%)	30 (75%)	20 (69%)	0.32
pN+ primary tumor (n, %)	45 (63%)	25 (63%)	18 (62%)	0.99
RAS mutant (n, %)	28 (39%)	18 (46%)	9 (30%)	0.22
Clinical risk score (median, IQR)	2 (2–3)	3 (2–3)	3 (2–3)	0.57
Tumor burden score (median, IQR)	8.3 (5.8–11.3)	9.1 (6.4–11.6)	7.3 (5.4–10.8)	0.17
Zone 1 (<3) (n, %)	1 (1%)	1 (2.5%)	0 (0%)	>0.99
Zone 2 (≥3 – 9)	41 (58%)	18 (46%)	21 (70%)	0.05
Zone 3 (≥9)	29 (41%)	20 (51%)	9 (30%)	0.09
Recurrent hepatic metastases (n, %)	7 (10%)	4 (10%)	2 (7%)	0.69
No. liver tumors at dx (median, range)	7 (1–30)	7 (1–30)	6 (2–30)	0.21
>10 liver tumors at dx (n, %)	21 (30%)	13 (33%)	8 (27%)	0.60
Size of largest liver tumor in cm (median, IQR)	2.6 (1.6–3.7)	2.6 (1.8, 5)	2.6 (1.9–3.4)	0.71
Bilobar disease (n, %)	67 (94%)	35 (90%)	29 (97%)	0.38
Extrahepatic disease (n, %)	14 (20%)	6 (15%)	6 (20%)	0.75
Peritoneum	1 (1%)	0 (0%)	1 (3%)	0.43
Retroperitoneal lymph nodes	5 (7%)	1 (2.5%)	3 (10%)	0.31
Lung	9 (13%)	5 (13%)	3 (10%)	>0.99
Multiple	1 (1%)	0 (0%)	3 (10%)	0.08
Receipt of neoadjuvant tx (n, %)	70 (99%)	40 (100%)	29 (100%)	>0.99
No. cycles of NAT (median, IQR)	8 (6–11)	9 (8–12)	8 (6–10)	0.14
Major hepatectomy (n, %)	21(30%)	8 (21%)	13 (43%)	0.06
Partial hepatectomy (n, %)	50 (70%)	32 (80%)	16 (55%)	0.04
Segments resected (median, IQR)	4 (3–5)	4 (3–5)	4 (3–5)	0.76
Concurrent hepatic ablation (n, %)	57 (80%)	34 (85%)	21 (72%)	0.23
Segments ablated (median, IQR)	3 (2–5)	3 (2–4)	3 (2–4)	0.71
Simultaneous colon resection (n,%)	38 (54%)	19 (49%)	18 (60%)	0.47
R0 resection, colectomy (n, %)	71 (100%)	39 (100%)	30 (100%)	>0.99
R0 resection, hepatectomy (n, %)	64 (90%)	33 (85%)	29 (97%)	0.13
Receipt of adjuvant therapy (n, %)	71 (100%)	39 (100%)	30 (100%)	>0.99
Floxuridine dose used in first 34 cases (n, %)	–	32 (80%)	2 (7%)	<0.001
Floxuridine dose use in last 35 cases (n, %)	–	8 (20%)	27 (93%)	<0.001

*IQR* interquartile range

**Fig. 1:**
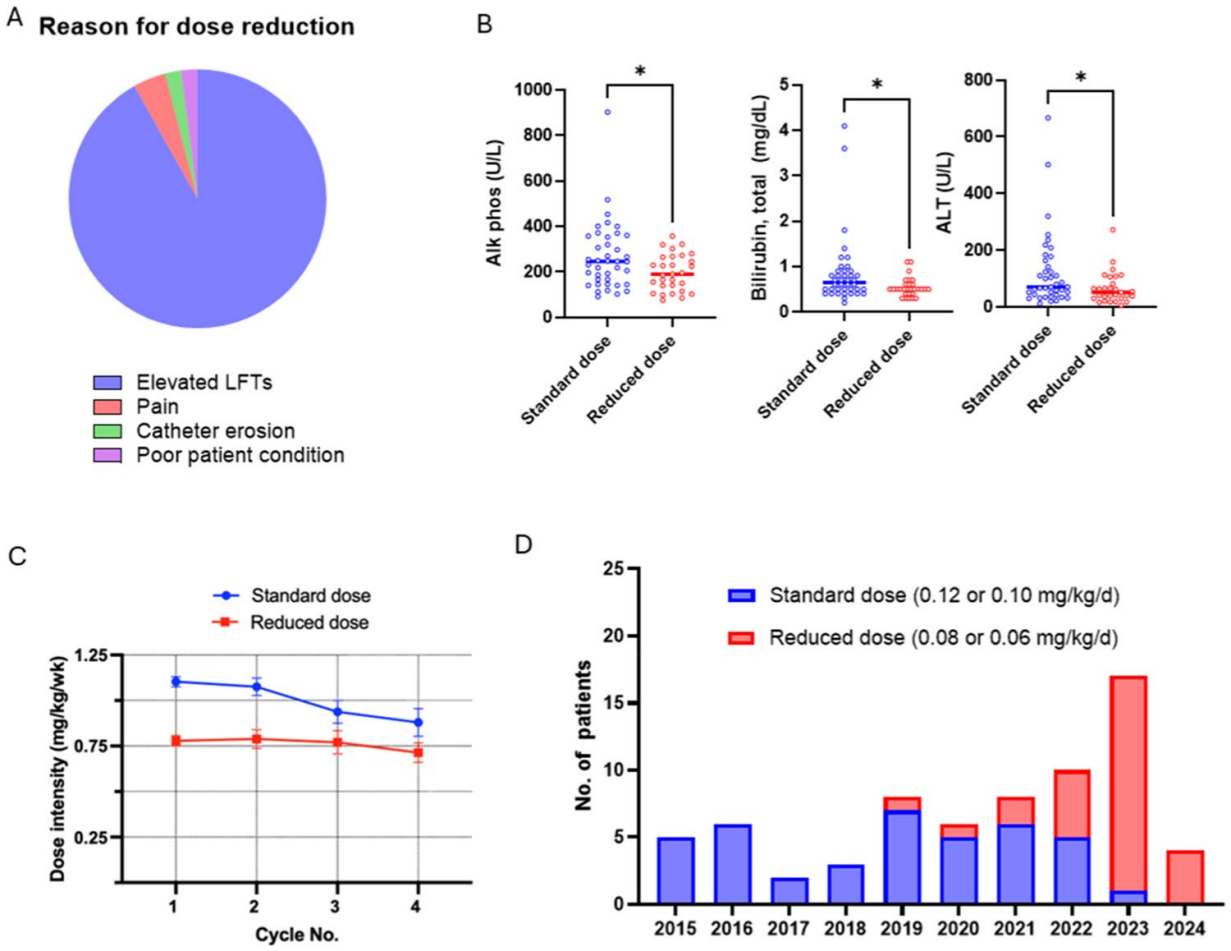
Dose alterations were primarily due to hepatobiliary toxicity. **A** Summarizes reason for first dose reduction. **B** Liver function tests (LFTs) in patients during treatment with either standard (0.12 or 0.1 mg/kg/day) or reduced dose (0.08 or 0.06 mg/kg/day). Symbol represents each patient; line represents mean. Star represents *p* < 0.05. **C** Dose intensity (mg/kg/week) of each cycle of drug received for the cohort of patients receiving standard or reduced starting dose. Symbol denotes mean and error bars denote standard error of the mean. **D** Trends in the starting floxuridine dose selected over time

Floxuridine dosing information was available for 69 patients (97%). Forty patients (58%) received the standard starting dose of floxuridine (0.12 or 0.1 mg/kg/day), and 29 patients (42%) received the reduced dose (0.08 or 0.06 mg/kg/day). Patients who were started on the standard dose were younger (*p* = 0.04), had higher rate of partial hepatectomy (*p* = 0.03), and mostly were treated during the first half of the study period (*p* < 0.001). Forty-eight patients (70%) required dose reductions during their treatment, mostly due to hepatobiliary toxicity (Fig. 1A; Table 2). More patients receiving the standard floxuridine dose required a dose reduction (82% vs. 53%, *p* = 0.04) and the magnitude of dose reduction was larger (2 vs. 1 mg/kg, *p* = 0.01) compared with patients receiving the reduced dose. However, patients who started on the standard dose received a higher total dose of floxuridine during their treatment course (8 vs. 6 mg/kg, *p* = 0.01).

**Table 2:** Floxuridine dosing characteristics and reasons for dose reduction

Starting dose	All patients N = 71	Standard dose n = 40	Reduced dose n = 29	*p*
Cycles received; median (range)	4 (2–6)	4 (2–6)	4 (2–5)	0.12
Dose reduction performed (patients); n (%)	49 (71%)	32 (82%)	16 (53%)	0.04
No. full dose cycles prior to dose alteration; median (range)	2 (1–3)	2 (1–3)	1.5 (1–2)	0.15
Original intended floxuridine dose (mg/kg); median (IQR)	8 (7–10)	10 (8–11)	7 (6–7)	<0.01
Total cumulative dose received (mg/kg); median (IQR)	6 (5–9)	8 (6–9)	6 (4–6)	<0.01
Cumulative dose reduction (mg/kg); (median, IQR)	1 (0–3)	2 (1–3)	1 (0–1.5)	0.01
Relative dose intensity (%); (median, IQR)	87 (72–100)	75 (66–90)	88 (74–100)	0.10
Relative dose intensity >80%, n (%)	38 (55%)	17 (43%)	21 (72%)	0.03
*Reason for dose reduction (n, %)*				
Chemical hepatitis	45 (90%)	28 (70%)	14 (48%)	0.04
Pain	2 (4%)	0	2 (7%)	0.99
Catheter erosion	1 (2%)	0	1 (3%)	0.99
Poor patient condition	1 (2%)	1 (2%)	0	0.99
Biliary sclerosis requiring stent	4 (6%)	4 (10%)	0	0.25

*IQR* interquartile range

The standard dose was associated with increased hepatobiliary toxicity compared to the reduced dose (Fig. 1B). Levels of alkaline phosphatase, bilirubin, and transaminases were significantly higher in patients treated with the standard dose. Most of the chemical hepatitis was transient, however biliary sclerosis requiring stent placement occurred in 4 patients, all of whom were started on the standard dose (10% biliary sclerosis rate for standard dose). Patients who were started on the standard dose received a larger total dose of floxuridine over the treatment course, but the dose decreased more significantly over time (Fig. 1C). Given the increased hepatobiliary toxicity associated with the standard dose, it was less commonly used over time and eventually discontinued (Fig. 1D).

We examined for risk factors associated with dose reduction (Table 3). Male sex was protective against dose reduction on multivariate analysis (odds ratio [OR] 0.26; 95% confidence interval [CI] 0.07–0.84). The extent of hepatic resection, duration of neoadjuvant therapy, volume of hepatic tumor burden, and patient risk factors such as obesity and hepatitis were not associated with dose reduction.

**Table 3:** Risk factors associated with dose reduction

Any dose reduction	Univariate (odds ratio [95% CI])	*p*	Multivariate (odds ratio [95% CI])	*p*
Age	1.00 [0.951–1.063]	0.87		
Male	0.30 –[0.0870.900]	0.04	0.26 [0.07–0.84]	0.04
BMI	0.94 [0.865–1.022]	0.16		
Obesity	0.59 [0.198–1.77]	0.33		
Hepatitis	0.85 [0.077–18.927]	0.89		
Clinical risk score	1.49 [0.783–3.000]	0.24		
CRS >2	1.70 [0.564–5.046]	0.34		
Major anatomic resection	2.25 [0.701–8.800]	0.20		
Tumor burden score	1.03 [0.935–1.171]	0.55		
Perioperative complication	0.72 [0.218–2.503]	0.60		
Synchronous resection	1.12 [0.396–3.127]	0.83		
Cycles of NAT (>8 cycles, median)	1.21 [0.390–3.607]	0.73		
Standard starting floxuridine dose	2.96 [1.044–8.877]	0.04	2.97 [1.01–9.31]	0.05

**Fig. 2:**
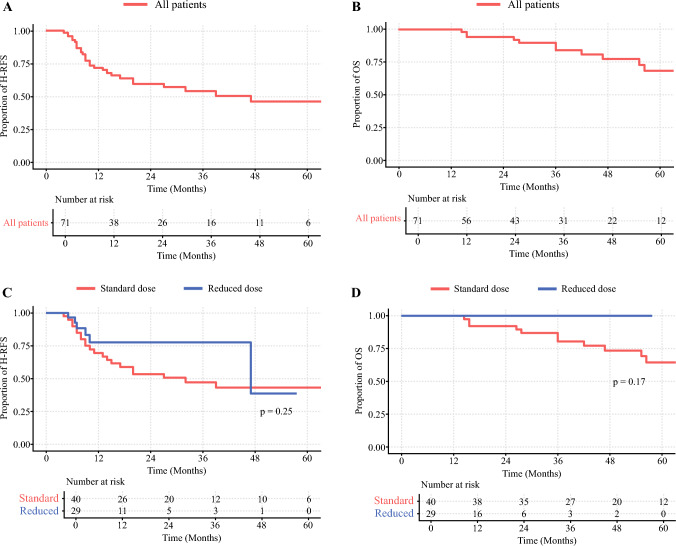
Survival was preserved with lower floxuridine starting dose. Hepatic recurrence-free **A** and overall survival **B** for entire cohort. Hepatic-recurrence free **C** and overall survival **D** for patients started on the standard or reduced floxuridine dose

Median follow up of the entire cohort, the standard and reduced dose group was 35, 58, and 14 months, respectively. For the entire cohort, the median OS was not met, while 3-year OS was 84% (Fig. 2A). The median hepatic-RFS was 3.9 years, while 3-year H-RFS was 54%. The reduced starting dose of floxuridine did not impact short-term hepatic recurrence-free or overall survival (Fig. 2C, D). Three-year OS was 86% and 100% for the standard and reduced dose group, respectively. Three-year H-RFS was 44% versus 83% for the standard and reduced dose group, respectively.

## Discussion

In this study, we examined the impact of the starting floxuridine dose on hepatobiliary toxicity and survival in patients with high-risk CRLM after complete hepatic resection. The reduced starting dose was less hepatotoxic, required fewer dose reductions, and did not negatively impact survival compared with standard dosing. Chemical hepatitis was transient in most patients; however, biliary sclerosis requiring endoscopic intervention occurred only in patients treated with standard dose at a rate of 10%. In this high-risk cohort, long-term hepatic-recurrence free survival and overall survival was prolonged and was not negatively impacted by reducing the floxuridine dose. These data support a reduced floxuridine dose (0.08 mg/kg/day) as the new standard for the adjuvant treatment of high-risk CRLM after complete resection.

The starting dose of floxuridine has ranged widely since it was first described in the 1980s for the treatment of unresectable CRLM.^9,10,14,25–28^ Depending on the indication, doses range from 0.1 to 0.5 mg/kg/day with higher doses associated with significant hepatic and biliary toxicity. Over time, a dose of 0.12 mg/kg/day was accepted as the standard dose in combination with modern systemic chemotherapy.^23,24,29^However, despite this reduced dose, 0.12 mg/kg/day of floxuridine is still poorly tolerated and is associated with frequent dose reductions and treatment disruptions. In one phase 2 clinical trial examining HAI floxuridine with systemic chemotherapy in patients with unresectable CRLM, the percentage of planned dose received was only 42% after 6 months of therapy.^30^ In efforts to improve tolerability, Schwantes et al. developed a modified dosing protocol of 0.06 mg/kg/day. Their elegant work demonstrates this reduced dose to be associated with fewer treatment disruptions, more consecutive floxuridine cycles, and a similar potential to convert patients with initially unresectable CRLM.^17^

Findings from our study indicate that patients may benefit from the reduced floxuridine dose of 0.08 mg/kg/day in the adjuvant treatment of resected CRLM. Like the treatment of unresectable CRLM, higher doses of floxuridine can have significant dose-limiting toxicity in the adjuvant setting. In a landmark adjuvant therapy trial, a starting floxuridine dose of 0.25 mg/kg/day was associated with significant hepatobiliary toxicity, with only a quarter of patients receiving more than 50% of planned dose.^9^ In our study, the standard dose of 0.12 mg/kg/day was more hepatotoxic with more severe chemical hepatitis and biliary sclerosis rate of 10%. The reduced dose of 0.08 mg/kg/day was better tolerated, with a lower incidence of chemical hepatitis requiring dose reductions and no biliary sclerosis as of this analysis. This is likely explained by a reduction in the total amount of drug received per cycle and over the treatment course. Factors we hypothesized to be associated with dose reductions, such as the extent of hepatic resection, tumor burden, cycles of chemotherapy, or other patient risk factors, such as hepatitis, cirrhosis, and obesity, were not significant. This may be due to limitations in sample size and challenges in data collection, such as underestimating the extent of hepatic resection/ablation from operative reports. Further research is needed with larger sample size to identify risk factors associated with hepatobiliary toxicity and dose reductions to improve tolerability of therapy.

The reduced floxuridine dose was associated with comparable oncological outcomes previously published in adjuvant floxuridine studies with a 5-year H-RFS of 47% and OS of 68%.^7,8^ Identifying the most tolerable dose with optimal oncologic outcomes is an important tenet in medical oncology. Sparing hepatic and biliary toxicity in the adjuvant treatment of these high-risk patients preserves future salvage therapies which are key to management of likely recurrences. Biliary sclerosis is an unforgiving complication that in many cases can preclude certain salvage therapies. Therefore, the authors now continue to use 0.08 mg/kg/day for the standard starting dose for all patients in the adjuvant setting.

This study has several limitations. First, the starting dose was selected based on the discretion of our medical oncologist and, given the study’s retrospective nature, is impacted by a selection bias. However, the higher standard starting dose (0.12 or 0.1) was uniformly selected in the beginning of the study period and then eventually uniformly changed to the reduced dose as standard (0.08 or 0.06). Second, the sample size is small and limited the multivariate analysis. Third, the more recent adoption of the reduced dose was associated with a more limited follow-up duration impacting our survival analysis, although we had sufficient follow-up to document preserved short-term survival in the reduced dose group. Finally, there was a slight difference in patient population between the standard and reduced dose group including more major resections with the standard dose, and could reflect changes in surgical practice over the study period.

In conclusion, as adjuvant floxuridine is increasingly utilized in the management of CRLM, careful consideration must be paid to minimizing hepatobiliary toxicity. In addition to limiting the dose received, hepatobiliary toxicity may limit future salvage therapies. This study provides evidence that the standard floxuridine dose of 0.12 mg/kg/day is associated with increased hepatobiliary toxicity without a clear survival benefit compared with a reduced dose in the adjuvant setting. Therefore, we consider the reduced dose of 0.08 mg/kg/day to be the new standard in the adjuvant treatment of completely resected CRLM.

## Disclosures

Yuman Fong—scientific advisor to Medtronics, Imugene, Vergent Biosciences, XDemics, Theromics, Eureka Biologics, Iovance Biotherapeutics, and Savato Health; receives royalties from Merck, Imugene. Marwan G. Fakih—AbbVie—Consultant; Adagene—Consultant; Bayer—Consultant; BMS—Consultant; Delcath Systems - Consultant; Eisai Inc.—Consultant; entos, Inc.—Advisory Board; Iterion Therapeutics—Consultant; Janssen Research & Development—Advisory Board; Merck—Consultant; Microbial Machines - Consultant; Mirati Therapeutics—Advisory/Consulting; Nouscom–Advisory Board; Pfizer—Consulting; Roche / Genentech—Advisory Board; Summit Therapeutics—Consulting; Taiho Oncology—Consulting; Tempus—Advisory Board; Totus Medicines—Consulting; Xenthera—Advisory Board; Xilio Therapeutics—Advisory Board; AgenusBio—Grant to Institution

## Supplementary Information

Below is the link to the electronic supplementary material.

Supplementary file1 (DOCX 121 kb)
