# Supplementary material for: Reduced Floxuridine Dose Limits Hepatobiliary Toxicity Without Negatively Impacting Survival After Resection of Colorectal Cancer Liver Metastases
Source: Ann Surg Oncol. 2025 Jul 22;32(13):9526–33. doi: 10.1245/s10434-025-17783-y (PMC12589368; doi:10.1245/s10434-025-17783-y)
Supplement: Supplementary file 1 — Supplementary file1 (DOCX 121 kb) [file 10434_2025_17783_MOESM1_ESM.docx]

**Supplemental Fig. 1** Hepatic tumor burden of cohort.

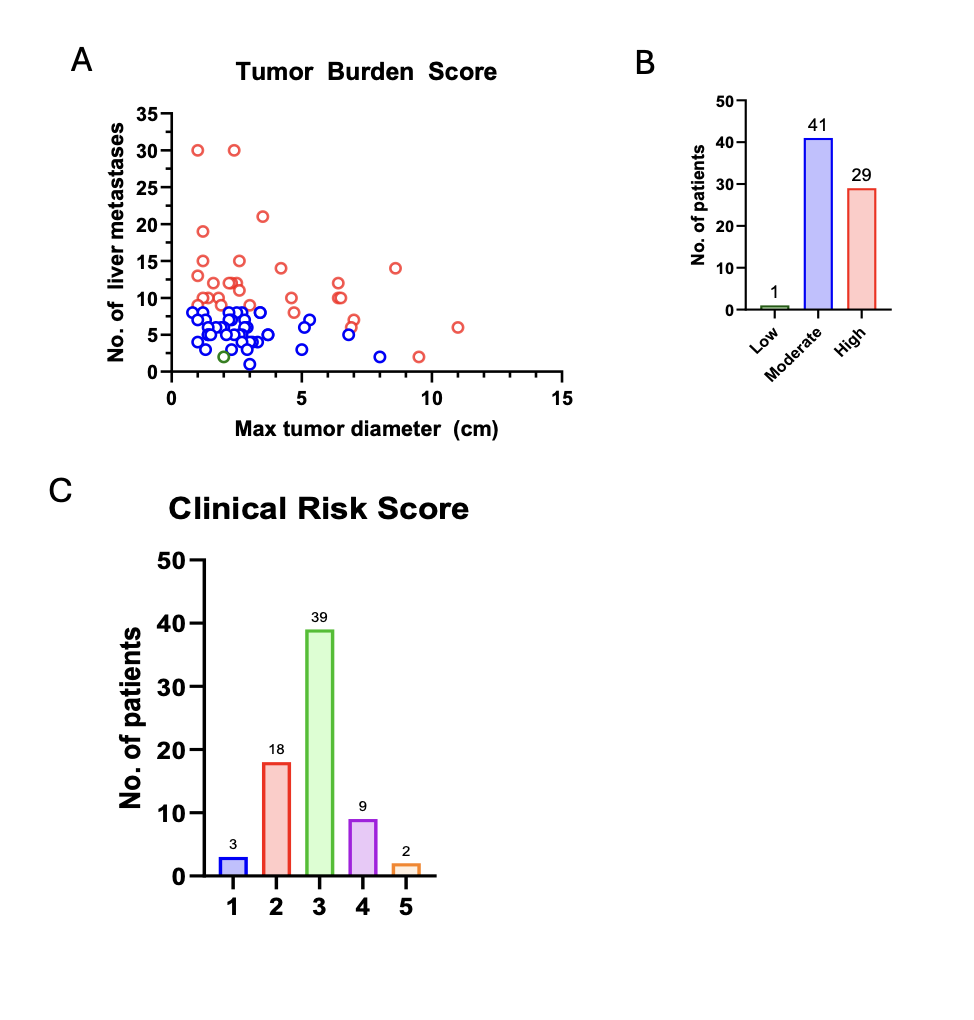


**A, B.** Tumor burden score of cohort. Symbol denotes patient. Green symbol = low tumor burden score; blue symbol = moderate tumor burden score; red symbol = high tumor burden score. **C.** Clinical risk score of cohort as described by Fong et al.
